# Neoadjuvant Chemohormonal Therapy Versus Pelvic Lymphadenectomy on Biochemical Recurrence in Patients with High- or Very-High-Risk Prostate Cancer Undergoing Robot-Assisted Radical Prostatectomy

**DOI:** 10.3390/diseases13040092

**Published:** 2025-03-23

**Authors:** Makoto Kawase, Satoshi Washino, Takato Nishino, Takeshi Yamasaki, Hajime Fukushima, Kosuke Iwatani, Tomoaki Miyagawa, Masaki Shimbo, Kojiro Ohba, Jun Miki, Keita Nakane, Takuya Koie

**Affiliations:** 1 Department of Urology, Graduate School of Medicine, Gifu University, Gifu 5011194, Japan; kawase.makoto.g5@f.gifu-u.ac.jp (M.K.); nakane.keita.k2@f.gifu-u.ac.jp (K.N.); 2 Department of Urology, Jichi Medical University Saitama Medical Center, Saitama 3300834, Japan; suwajiisan@jichi.ac.jp (S.W.); sh2-miya@jichi.ac.jp (T.M.); 3 Department of Urology, St. Luke’s International Hospital, Tokyo 1048560, Japan; tn5mrc1121@gmail.com (T.N.); mashimbo@luke.ac.jp (M.S.); 4 Department of Urology, Graduate School of Medicine, Osaka Metropolitan University, Osaka 5458585, Japan; urology-yamasaki@outlook.jp; 5 Department of Urology and Renal Transplantation, Nagasaki University Hospital, Nagasaki 8528501, Japan; h.fukushima@nagasaki-u.ac.jp (H.F.); ohba-k@nagasaki-u.ac.jp (K.O.); 6 Department of Urology, The Jikei University School of Medicine, Kashiwa Hospital, Kashiwa 2778567, Japan; k.iwatani.urology@gmail.com (K.I.); junmiki.jikei@gmail.com (J.M.)

**Keywords:** prostate cancer, high- and very-high-risk prostate cancer, robot-assisted radical prostatectomy, neoadjuvant chemo-hormonal therapy, extended pelvic lymph node dissection

## Abstract

Background/Objectives: The effectiveness of robot-assisted radical prostatectomy (RARP) with extended pelvic lymph node dissection (ePLND) in improving oncological outcomes for patients with high- or very-high-risk prostate cancer (HR/VHR-PCa) remains a subject of debate. This study aimed to compare the efficacy of neoadjuvant chemohormonal therapy (NCHT) and ePLND in reducing biochemical recurrence (BCR) in patients with HR/VHR-PCa undergoing RARP. Methods: This retrospective, multicenter cohort study included 1182 patients with HR/VHR-PCa who underwent RARP at six Japanese institutions. Patients were stratified into three groups: those who received NCHT followed by RARP without ePLND (Group 1), those who received neoadjuvant hormonal therapy (NHT) followed by RARP with ePLND (Group 2), and those who underwent RARP with ePLND (Group 3). The primary endpoint was the rate of BCR, while the secondary endpoint was biochemical recurrence-free survival (BRFS) following RARP. Results: Of the 1182 patients, 154 patients were included in Group 1, 97 patients were included in Group 2, and 470 patients were included in Group 3. By the end of the follow-up period, 243 patients (33.8%) had experienced BCR, 27 (3.7%) had progressed to castration-resistant prostate cancer, and 5 (0.7%) had died from PCa. Over a median follow-up period of 41.4 months, BCR occurred in 16.5% of patients in Group 1, 36.1% of patients in Group 2, and 38.9% in Group 3 (*p* < 0.001). The 3-year BRFS rate was 63.6% in Group 1, 53.1% in Group 2, and 63.9% in Group 3. Conclusions: The findings of this study indicate that NCHT in patients with HR/VHR-PCa undergoing RARP without ePLND may reduce the risk of postoperative BCR compared to those undergoing RARP with ePLND.

## 1. Introduction

High-risk (HR-) or very high-risk (VHR-) prostate cancers (PCas) are associated with an increased likelihood of biochemical recurrence (BCR), clinical recurrence, castration-resistant PCa (CRPC), and PCa-related mortality following definitive treatment [1,2]. According to the 2014 International Society of Urologic Pathology (ISUP) guidelines, HR-PCa is defined by one or more of the following criteria: an initial prostate-specific antigen (PSA) level > 20 ng/mL, clinical T stage (cT) ≥ 3a, or grade group (GG) ≥ 4 [3]. Similarly, VHR-PCa is characterized by the presence of at least one of the following factors: cT3b or T4 in the biopsy specimen, primary Gleason pattern 5, or at least five biopsy cores containing GG4 or GG5 disease [4]. The National Comprehensive Cancer Network (NCCN) guidelines recommend several definitive treatment options for HR/VHR-PCa, including radical prostatectomy (RP) with pelvic lymph node dissection (PLND), external beam radiotherapy (EBRT) combined with androgen deprivation therapy (ADT), or a trimodal approach consisting of ADT, brachytherapy, and EBRT [4]. However, it is well recognized that many patients with HR/VHR-PCa harbor micrometastatic disease undetectable by conventional imaging modalities, leading to a high incidence of BCR following surgery alone, particularly in cases of robot-assisted radical prostatectomy (RARP) with PLND [5]. Although several recent studies have highlighted the diagnostic value of PLND in providing accurate histopathological staging, the therapeutic benefits and optimal extent of dissection remain controversial [6,7,8]. Additionally, PLND has not been consistently associated with improved oncological outcomes, particularly biochemical recurrence-free survival (BRFS) [6,7,8]. Furthermore, extended PLND (ePLND) has been linked to an increased risk of perioperative complications, including bleeding, lymphocele formation, thromboembolism, and prolonged operative time [9].

To enhance oncological outcomes, multidisciplinary treatment strategies incorporating surgical and neoadjuvant therapies have been explored [10,11,12,13,14,15,16]. Trials evaluating neoadjuvant hormone therapy (NHT) with androgen receptor signaling inhibitors have reported improved BCR rates and metastasis-free survival in patients undergoing surgery after NHT compared to those undergoing RP or RARP alone [10]. However, a multicenter prospective randomized controlled trial assessing the efficacy of NHT in PCa failed to demonstrate a statistically significant improvement in oncological outcomes [17]. Thus, current guidelines do not recommend routine NHT before surgery [4,18]. In contrast, neoadjuvant chemohormonal therapy (NCHT), which combines ADT with cytotoxic anticancer agents, has shown potential benefits in HR/VHR-PCa, with studies reporting improved BRFS and overall survival (OS) when combined with surgical treatment [12,13,14,15,16].

Given these findings, we hypothesized that NCHT could be a viable alternative to ePLND in patients with HR/VHR-PCa undergoing RARP, offering both oncological safety and reduced perioperative morbidity. Therefore, this study aimed to evaluate the impact of NCHT on oncological outcomes and surgical safety in this patient population.

## 2. Materials and Methods

### 2.1. Ethics Statement and Patient Characteristics

This study was approved by the Institutional Review Board of Gifu University (approval number: 2022–AL0574) and the respective ethics committees of all participating institutions. Owing to the retrospective nature of the study, informed consent was obtained on an opt-out basis. According to the Japanese Ethical Guidelines and institutional regulations, explicit written consent is not required for retrospective observational studies, as data are collected from existing records and publicly available sources. Additional details about this study, available in Japanese, can be accessed at https://rinri.med.gifu-u.ac.jp/esct/publish_document.aspx?ID=2570 (accessed on 1 January 2025). 

A total of 1182 patients with HR/VHR-PCa who underwent RARP at six Japanese centers between May 2014 and December 2022 were included in this retrospective, multicenter cohort study. The collected preoperative clinical and perioperative parameters included age, height, weight, body mass index (BMI), initial serum PSA level, prostate volume (PV), biopsy Gleason grade group (bGG), clinical stage, percentage of positive biopsy cores, NCCN risk stratification [4], use and duration of neoadjuvant and/or adjuvant therapy, adverse events (AEs) related to pharmacological treatment, console time, estimated blood loss (EBL), presence of PLND, nerve-sparing surgery status, and perioperative complications. Tumor staging was determined according to the American Joint Committee on Cancer Staging Manual, 8th Edition [19]. Pathological evaluation focused on tumor characteristics, including the T and N stages of the surgical specimen, the number of removed lymph nodes, the pathological Gleason grade group (pGG), the presence of extraprostatic extension, seminal vesicle invasion, lymphovascular invasion (LVI), and surgical resection margin (RM) status. The extent of PLND varied among patients and was categorized as either limited (restricted to the obturator lymph nodes) or extended (including lymph node dissection up to the intersection of the common iliac artery and ureter, with or without presacral lymph nodes) [20]. The necessity and extent of PLND were determined based on the surgeon’s discretion and institutional protocols. Perioperative complications were classified using the Clavien–Dindo grading system [21].

All patients underwent preoperative imaging, including computed tomography of the chest and pelvis and magnetic resonance imaging of the prostate, to assess local tumor invasion, lymph node metastases, and distant metastases. Patients were stratified into three groups: (1) those who received NCHT consisting of a gonadotropin-releasing hormone (GnRH) antagonist and tegafur-uracil (UFT) followed by RARP without PLND (Group 1), (2) those who received NHT consisting of a GnRH agonist/antagonist and/or bicalutamide followed by RARP with ePLND (Group 2), and (3) those who underwent RARP with ePLND (Group 3). In Group 1, patients received a GnRH antagonist at an initial dose of 240 mg, followed by a maintenance dose of 80 mg per month, along with oral UFT at 300 mg/day for a minimum of three months prior to RARP. In Group 2, the combination and duration of NHT were determined based on the surgeon’s discretion and institutional protocols. The NCHT-related AEs were evaluated using the Common Terminology Criteria for Adverse Events, version 5.0 [22].

### 2.2. Follow-Up Schedule

Serum PSA and testosterone levels were measured every three months after RARP for all enrolled patients. In Group 1, GnRH antagonist and UFT therapy were discontinued following RARP. BCR was defined as a serum PSA level exceeding 0.2 ng/mL on at least two consecutive assessments. If PSA levels did not decrease below 0.2 ng/mL postoperatively, the date of RARP was considered the date of BCR [23].

### 2.3. Histopathological Analysis

All prostate specimens were evaluated according to the 2005 International Society of Urologic Pathology consensus guidelines [24] using whole-layer staining. The prostate apex was sectioned perpendicularly along the prostatic urethra, and residual prostate tissue was sectioned at 3–5 mm intervals perpendicular to the urethral axis for detailed histopathological assessment.

### 2.4. Endpoints and Statistical Analysis

The primary endpoint of this study was BRFS. Secondary endpoints included surgical outcomes, perioperative complications, and the safety of NCHT prior to RARP. Statistical analyses were conducted using JMP Pro 17 (SAS Institute Inc., Cary, NC, USA). Continuous variables were summarized using medians and interquartile ranges (IQR), whereas categorical variables were reported as absolute values and percentages. Kaplan–Meier survival analysis was performed to evaluate BRFS following RARP, with the log-rank test used to assess associations between BCR and covariates. A *p*-value of <0.05 was considered statistically significant in all analyses.

## 3. Results

### 3.1. Patients and Characteristics

Of the 1182 patients with HR/VHR-PCa in the database, 721 were included in this study. In Group 1, with 162 patients, we excluded eight patients who had received PLND, resulting in 154 cases. In Group 2, with 187 patients, 81 who had not undergone PLND or had received limited PLND (L-PLND) and 9 patients who received only bicalutamide were excluded, resulting in 97 cases. In Group 3, with 833 patients, 363 patients who had received no PLND or L-PLND were excluded, resulting in 470 cases (Group 3). A total of 721 patients with HR/VHR PCa were selected. The preoperative clinical characteristics of patients with HR/VHR-PCa are presented in Table 1.

### 3.2. Surgical and Oncological Outcomes

Perioperative and pathological outcomes are shown in Table 2. The median follow-up duration for all enrolled patients was 41.4 months (IQR: 17.0–69.5 months).

**Figure 1:**
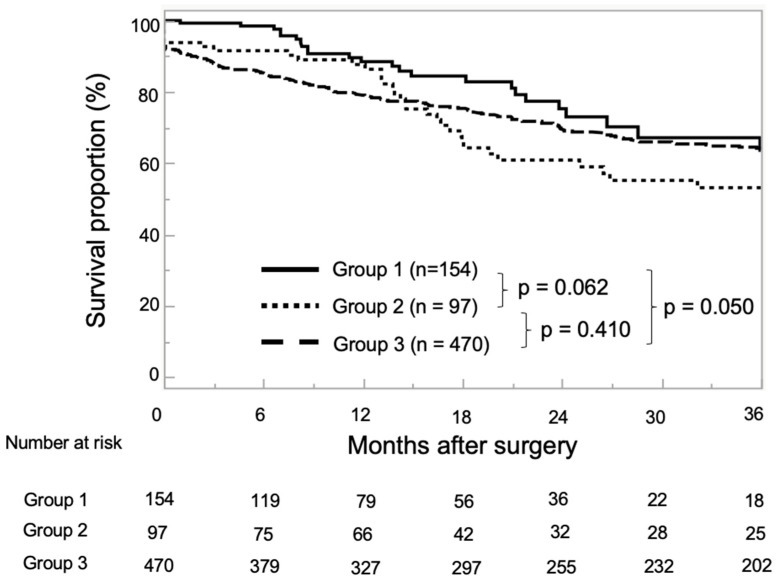
Kaplan–Meier estimates for neoadjuvant chemohormonal therapy (NCHT) followed by robot-assisted radical prostatectomy (RARP) without pelvic lymph node dissection (PLND) (Group 1), neoadjuvant hormonal therapy (NHT) followed by RARP with extended PLND (ePLND) (Group 2), and RARP with ePLND (Group 3) for biochemical recurrence-free survival (BRFS). The 1- and 3-year BRFS rates were 88.5% and 63.6% in Group 1, 86.3% and 53.1% in Group 2, and 78.8% and 63.9% in Group 3, respectively.

At the end of the observation period, 243 patients (33.8%) had experienced BCR, 27 (3.7%) had progressed to CRPC, and 4–5 (0.7%) had died from PCa. During follow-up, BCR was found to have occurred in 16.5% of Group 1, 36.1% of Group 2, and 38.9% of Group 3 (*p* < 0.001), while CRPC was observed in 2.0% of Group 1, 5.2% of Group 2, and 4.0% of Group 3 (*p* = 0.324). There were only four PCa-related deaths, one in Group 2, and in three in Group 3. The median BRFS for all enrolled patients was 20.9 months (IQR: 7.4–47.6 months). The 1- and 3-year BRFS rates were 88.5% and 63.6% in Group 1, 86.3% and 53.1% in Group 2, and 78.8 and 63.9% in Group 3, respectively (Figure 1).

### 3.3. Perioperative Complications

Table 3 summarizes the perioperative complications associated with RARP. The ePLND group (Group 2 and 3) had a higher incidence of perioperative complications compared to the NCHT group (Group 1), particularly in terms of edema and lymphocele formation.

### 3.4. Safety of Neoadjuvant Chemohormonal Therapy with Combined GnRH Antagonist and UFT

The median duration of treatment for patients who received NCHT was 4.3 months (IQR: 3.8–5.1 months). Of the 154 patients who received NCHT, 38 (24.7%) developed AEs of any grade. The most common NCHT-related AE was hepatotoxicity, occurring in thirty-two cases (20.8%) of all grades, including two (1.3%) cases of grade 3 severity. Other AEs included rash and fatigue in two patients (1.3%) and anemia and gynecomastia in one patient (0.6%). The GnRH antagonist was continued in all patients, although 13 (8.4%) patients either discontinued or reduced their UFT dose owing to AEs.

### 3.5. Recovery of Testosterone Levels After NCHT

**Figure 2:**
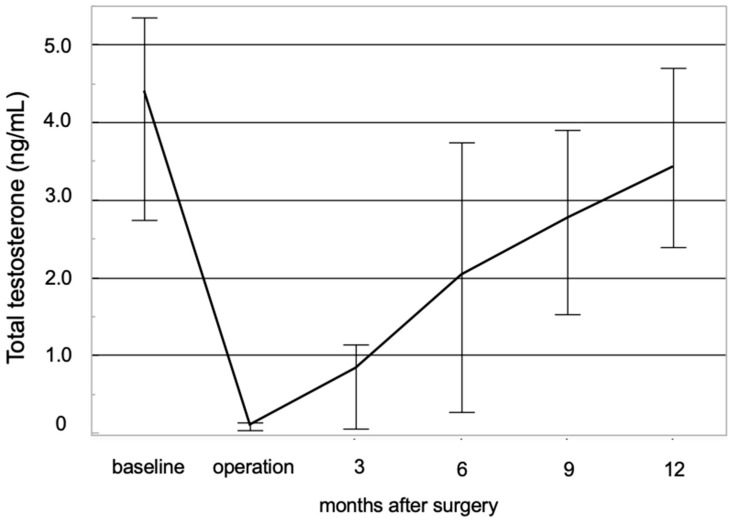
Chronological changes in the total serum level of testosterone have been studied. Total serum testosterone levels in patients receiving neoadjuvant chemohormonal therapy demonstrated a prompt decline following treatment initiation, subsequently exhibiting a gradual recovery trajectory after surgery.

In the NCHT group, serum total testosterone (TST) levels were measured in both the preoperative and postoperative periods. The chronological changes in serum testosterone levels are illustrated in Figure 2. The median TST value at the initiation of NCHT was 4.3 ng/mL (IQR: 2.7–5.4 ng/mL). TST levels rapidly declined following the initiation of NCHT, remaining below the castration threshold of 0.5 ng/mL until RARP in all patients. The median TST at RARP was 0.07 ng/mL (IQR: 0.03–0.13 ng/mL). Postoperatively, the median TST levels at 6 and 12 months were 1.3 ng/mL (IQR: 0.3–3.7 ng/mL) and 3.3 ng/mL (IQR: 2.4–4.7 ng/mL), respectively.

## 4. Discussion

HR/VHR-PCa is widely recognized as the most malignant form of PCa, often leading to early BCR after definitive treatment, followed by distant metastasis and cancer-related mortality [1,2]. Despite adopting various therapeutic strategies, there is no consensus on the optimal multidisciplinary approach for HR/VHR-PCa management [4]. Surgical therapy, particularly RARP, is considered a viable treatment option for select patients; however, its effectiveness in controlling HR/VHR-PCa remains unclear [4,18,25]. Although robotic surgery is increasingly utilized in PCa management, achieving oncological control in HR/VHR-PCa through RARP alone remains challenging [26]. A multicenter retrospective study involving 2670 patients with PCa who underwent RARP demonstrated that the 2-year BRFS rates were 97.1% and 91.8% for patients with low- and intermediate-risk PCa, respectively [25]. Conversely, the BRFS rate for HR/VHR-PCa patients was 76.6%, with a significantly higher incidence of BCR (*p* < 0.001) [26]. These findings highlight the need for a multidisciplinary treatment approach that combines various therapeutic modalities to improve oncological outcomes in patients with HR/VHR-PCa.

ePLND is believed to play a pivotal role in accurate PCa staging and detecting regional lymph node involvement (LNI), potentially improving oncological outcomes in cases of locoregional LNI [27,28]. According to the European Urological Association guidelines, ePLND is recommended for patients with HR-PCa or intermediate-risk PCa when the probability of LNI exceeds 5%, as determined by the Briganti nomogram [18,29]. However, previous studies have failed to demonstrate a significant oncological benefit of ePLND over L-PLND in patients with PCa undergoing RARP [6,7,8,9]. A prospective single-center phase III trial involving patients with intermediate- and HR-PCa randomized them 1:1 to receive bilateral L-PLND (obturator lymph nodes) or ePLND (obturator, external iliac, hypogastric, common iliac, and presacral lymph nodes) [6]. The median BRFS was not reached in the ePLND group and was 61.4 months in the L-PLND group, with no statistically significant difference between the two groups (hazard ratio [HR]: 0.91; 95% confidence interval [CI]: 0.63–1.32; *p* = 0.60) [6]. However, a subgroup analysis of patients who underwent ePLND revealed that those with a bGG of 3–5 had a superior BRFS compared to those with a GG of ≤2 (HR: 0.33; 95% CI: 0.14–0.74; *p* < 0.001) [6]. Similarly, a single-center randomized trial of 1440 patients undergoing RP with PLND, including 700 in the L-PLND group and 740 in the ePLND group, found no significant difference in BRFS at a median follow-up of 3.1 years (HR: 1.04; 95% CI: 0.93–1.15; *p* = 0.50) [7]. Furthermore, a multicenter retrospective study of 3195 PCa patients who underwent RARP found no significant BRFS differences between patients who underwent PLND and those who did not at all risks based on the NCCN risk classification, following a 1:1 PSM (605 patients in each group) (2-year BRFS: 94.3% vs. 95.8%; *p* = 0.855) [8]. However, ePLND is associated with a higher incidence of serious surgery-related complications compared to L-PLND [9]. Studies on ePLND-related complications have reported that lymphocele formation is the most frequent postoperative complication (90.6%), with its occurrence independently associated with the extent of PLND (HR: 0.41; 95% CI: 0.27–0.63; *p* < 0.01) [9,30]. Additional complications such as symptomatic lymphocele (2.5%), thromboembolism (0.3–0.5%), and sensory and motor disturbances (0.1%) have also been reported [30]. Given these risks, PLND, particularly ePLND, should be performed selectively, balancing the potential oncological benefits against the risk of postoperative PLND-related complications.

Owing to the complexity of HR/VHR-PCa management with RARP and ePLND alone, attention has shifted toward neoadjuvant or adjuvant therapies. A prospective study of 176 patients with PCa and cT3 disease found that BCR occurred in 64 patients (36%) who received NHT before surgery compared to 102 patients (64%) who underwent RP alone [1]. Among the total number of enrolled patients, 84 (48%) exhibited BCR, with a mean follow-up duration of 6.4 years [1]. The median time from surgery to BCR was 4.6 years [1]. However, NHT did not significantly reduce BCR incidence compared to RP alone [1]. In a retrospective study of 100 patients with localized PCa, 36 (36%) developed BCR at a median of 16.2 months [31]. Patients with HR/VHR-PCa exhibited significantly shorter time to BCR than low- and intermediate-risk PCa patients (median: 34.7 vs. 53.1 months; *p* = 0.04) [31]. Among patients with HR/VHR-PCa, those receiving adjuvant therapy had a significantly longer time to BCR than those who did not (median: 19.6 vs. 60.5 months; *p* = 0.03) [31]. However, median BCRs were comparable for patients with HR/VHR-PCa receiving adjuvant therapy and those with low- and intermediate-risk PCa (*p* = 0.69) [31]. NCHT with ADT and estramustine (EMP) has significantly improved 5-year BRFS (90.4% vs. 65.8%; *p* < 0.001) and OS (98.5% vs. 89.5%; *p* = 0.021) compared to RP alone [12,16]. The 2- and 5-year BRFS rates for patients with HR/VHR-PCa treated with NCHT combined with ADT, EMP, and docetaxel (DOC) were 69.2% and 60.1%, respectively [13]. However, grade ≥3 AEs were reported in 8.3% of patients, with 33.3% requiring dose adjustments for DOC or EMP [13]. Three patients developed deep vein thrombosis, with one succumbing to pulmonary embolism [13]. A prospective study of 21 patients with HR/VHR-PCa receiving neoadjuvant ADT + DOC reported a 5-year BRFS rate of 57.1%, with HR-PCa patients showing better outcomes (76.9% vs. 25.0%; *p* = 0.023) [32]. Our previous studies demonstrated that neoadjuvant ADT + UFT improved BRFS in patients with HR/VHR-PCa undergoing RARP [14,15]. In the current study, a comparison of Group 2 and Group 3 revealed an absence of an additive effect of NHT. Nevertheless, neoadjuvant ADT + UFT tended to reduce BCR incidence compared to ePLND in patients with HR/VHR-Ca undergoing RARP between Group 1 and 3. As ePLND has not been shown to improve oncological outcomes in randomized controlled trials, it may be more effective to perform NCHT rather than ePLND to improve oncological outcomes. These findings suggest that ePLND may be omitted in select patients receiving neoadjuvant ADT + UFT, providing a potentially less invasive yet effective approach to HR/VHR-PCa management.

This study identified several limitations in the research. First, as a multicenter, retrospective cohort study, potential biases may have arisen owing to variations in patient backgrounds, diagnostic accuracy, treatment strategies, and surgical techniques across participating centers. Second, the relatively short follow-up period limited our abilities to assess metastasis-free survival and radiographic progression-free survival, the incidence of CRPC, OS, and PCa-specific mortality. Third, prostate biopsies and surgical specimens were not reevaluated by a single urologic pathologist, potentially introducing variability in GG classification, RM status, and LVI assessment. Finally, in evaluating BRFS in the NHT and NCHT groups, the effect of TST recovery should be considered, necessitating cautious interpretation of the study findings.

## 5. Conclusions

This study suggests that NCHT followed by RARP without ePLND may provide improved BRFS compared to RARP with ePLND in patients with HR/VHR-PCa regardless of NHT. Further prospective clinical trials with longer follow-up periods are requisite to determine the long-term survival benefits of this treatment approach.

## Figures and Tables

**Table 1:** Patient characteristics.

	Group 1 (n = 154)	Group 2 (n = 97)	Group 3 (n = 470)	*p*-Value ^†^
Age (year, median, IQR)	72 (69–75)	69 (65–74)	68 (63–73)	<0.001
BMI (median, IQR)	23.6 (21.8–25.6)	24.0 (21.7–25.7)	23.8 (22.0–25.7)	0.411
initial PSA (ng/mL, median, IQR)	10.5 (6.5–19.6)	17.9 (9.3–31.7)	9.1 (5.9–14.7)	<0.001
Prostate volume (mL, median, IQR)	30 (22–40)	27 (21–39)	27 (21–35)	0.486
bGG (number, %)				0.005
1	0 (0)	3 (3.1)	9 (1.9)	
2	7 (4.6)	3 (3.1)	24 (5.1)	
3	8 (5.2)	13 (13.4)	23 (4.9)	
4	71 (46.1)	37 (38.1)	250 (53.2)	
5	68 (44.2)	41 (42.3)	164 (34.9)	
Clinical T stage (number, %)				0.106
1	6 (3.9)	6 (6.3)	36 (7.7)	
2	115 (75.2)	59 (61.5)	324 (68.9)	
3a	22 (14.4)	21 (21.9)	88 (18.7)	
3b	10 (6.5)	10 (10.4)	22 (4.7)	
Percent of positive core (%, median, IQR)	42 (25–59)	50 (33–75)	33 (19–50)	<0.001
NCCN risk classification (number, %)				0.079
High	118 (76.6)	68 (70.1)	378 (80.4)	
Very high	36 (23.4)	29 (29.9)	92 (19.6)	
Follow-up period (month, median, IQR)	14 (6–27)	31 (15–53)	56 (32–79)	<0.001

IQR, interquartile range; BMI, body mass index; PSA, prostate specific antigen; bGG, biopsy grade group; NCCN, the National Comprehensive Cancer Network. ^†^ Kruskal–Wallis test; Fisher’s exact test.

**Table 2:** Surgical and pathological outcomes.

	Group 1 (n = 154)	Group 2 (n = 97)	Group 3 (n = 470)	*p*-Value ^†^
Console time (min, median, IQR)	111 (82–126)	231 (189–273)	262 (222–293)	<0.001
Estimated blood loss (mL, median, IQR)	20 (5–50)	100 (50–250)	100 (50–210)	<0.001
Pathological T stage (number, %)				0.002
≤2	106 (68.8)	41 (42.3)	279 (59.6)	
≥3	47 (30.5)	49 (50.5)	189 (40.2)	
Pathological N stage (number, %)	Not applicable	28 (28.9)	74 (15.7)	Not evaluated
Lymph node count (number, median, IQR)	Not applicable	18 (11–24)	19 (14–25)	Not evaluated
RM positive (number, %)	34 (22.8)	30 (33.0)	126 (28.8)	0.193
LVI positive (number, %)	32 (20.9)	30 (31.6)	163 (34.8)	0.005
Adjuvant therapy (number, %)				0.029
Radiation therapy	7 (4.6)	2 (2.1)	12 (2.6)	
Hormonal therapy	2 (1.3)	9 (9.3)	26 (5.5)	
Both	0 (0)	1 (1.0)	11 (2.3)	

IQR, interquartile range; RM, resect margins; LVI, lymphovascular invasion. ^†^ Kruskal–Wallis test; Fisher’s exact test.

**Table 3:** RARP-related perioperative complication according to the Clavien–Dindo calcification.

Complication (Number, %)	Group 1 (n = 154)	Group 2 (n = 97)	Group 3 (n = 470)	*p*-Value *
All	9 (5.8)	16 (16.5)	96 (18.8)	<0.001
Edema	0 (0)	3 (3.1)	26 (5.5)	0.009
Anastomotic leakage	3 (1.9)	1 (1.0)	12 (2.6)	0.630
Lymphocele	0 (0)	1 (1.0)	12 (2.6)	0.098
Hernia	3 (1.9)	6 (6.2)	11 (2.3)	0.086
Urinary retention	0 (0)	0 (0)	6 (1.3)	0.199
Surgical site infection	0 (0)	0 (0)	4 (0.9)	0.342
Ileus	1 (0.6)	1 (1.0)	4 (0.9)	0.946
Urine bleeding	1 (0.6)	0 (0)	2 (0.4)	0.738
Others	2 (1.3)	4 (4.1)	19 (4.0)	0.252

* Fisher’s exact test.

## Data Availability

The data presented in this study are available from the corresponding author upon request. The data are not publicly available due to privacy and ethical reasons.
